# Physeal‐sparing anterior cruciate ligament reconstruction provides better initial joint biomechanics than complete transphyseal reconstruction in an early adolescent porcine model

**DOI:** 10.1002/jeo2.70289

**Published:** 2025-10-06

**Authors:** Yukun Zhang, Kaan Gurbuz, Logan Opperman, Jeffrey T. Spang, Matthew B. Fisher

**Affiliations:** ^1^ Lampe Joint Department of Biomedical Engineering North Carolina State University & University of North Carolina at Chapel Hill Raleigh North Carolina USA; ^2^ Comparative Medicine Institute North Carolina State University Raleigh North Carolina USA; ^3^ Department of Orthopedics and Traumatology, Kayseri Medical Faculty University of Health Sciences Kayseri Turkey; ^4^ Department of Statistics North Carolina State University Raleigh North Carolina USA; ^5^ Department of Orthopaedics University of North Carolina at Chapel Hill Chapel Hill North Carolina USA

**Keywords:** ACL reconstruction, biomechanics, knee, paediatrics, robotics

## Abstract

**Purpose:**

The aim of this study was to compare initial joint kinematics and tissue forces following complete transphyseal, partial transphyseal and physeal‐sparing anterior cruciate ligament (ACL) reconstruction (ACLR) in an early adolescent porcine model.

**Methods:**

Eighteen early adolescent porcine knees were tested using a six‐degree‐of‐freedom robotic testing system at 40° and 60° of flexion. An 80 N anterior‐posterior (AP) force, 120 N compression force and 4 N m varus‐valgus torque were applied to the tibia in intact, ACL transected and ACLR states. Complete transphyseal, partial transphyseal, and physeal‐sparing surgical techniques were performed (*n* = 6 legs/technique). Kinematics under applied loads were recorded to assess joint stability and compared across each state. Individual tissue forces were calculated using the principle of superposition. For comparisons between surgical techniques, both joint stability and tissue forces were normalized to intact control data from the same joints.

**Results:**

At 40° of flexion, the increase in AP tibial translation following physeal‐sparing ACLR was 3.8 mm smaller than the complete transphyseal (*p* = 0.02). Under anterior tibial loading, the anterior force taken by the reconstructed ACL graft significantly decreased similarly following reconstruction by each technique, which shifted to the medial collateral ligament (MCL). The increase in anterior MCL forces following complete transphyseal reconstruction was 283% higher than that after physeal‐sparing reconstruction (*p* = 0.04). Under valgus torque, the change in compression force in the lateral meniscus following physeal‐sparing reconstruction was 82% smaller than that after partial transphyseal reconstruction (*p* = 0.02). At 60° of flexion, the average increase in ATT under compression following partial transphyseal and physeal‐sparing reconstruction was 5.0 and 6.9 mm smaller than following complete transphyseal reconstruction (*p* ≤ 0.001 for each).

**Conclusion:**

In early adolescent porcine joints, the physeal‐sparing technique led to better initial joint stability with less anterior force shifted to the MCL compared to the complete transphyseal technique.

## Graphical abstract

In early adolescent porcine joints, the physeal‐sparing anterior cruciate ligament reconstruction technique led to better initial joint stability and tissue function compared to the complete transphyseal technique.

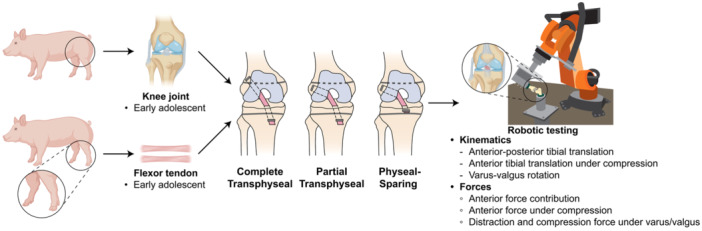

**Abbreviations**
ACL: anterior cruciate ligament
ACLR: ACL reconstruction
ACLT: ACL transection
AM bundle: anteromedial bundle
AP: anterior‐posterior
APTT: anterior‐posterior tibial translation
ATT: anterior tibial translation
CSA: cross‐sectional area
IT band: iliotibial band
LCL: lateral collateral ligament
LMEN: lateral meniscus
MCL: medial collateral ligament
MMEN: medial meniscus
PBS: phosphate‐buffered saline
PL bundle: posterolateral bundle
VV: varus‐valgus

## INTRODUCTION

The rate of paediatric anterior cruciate ligament (ACL) injuries has been increasing steadily [14, 31]. Relative to nonoperative management, surgical treatment of paediatric ACL injuries has demonstrated the ability to restore knee stability and reduce the risk of chondral and meniscal injuries, along with a higher rate of return to sports [2, 15, 16]. However, high re‐rupture rates and growth disturbances, including angular deformity and limb‐length discrepancy, have been reported [7, 19, 20, 46], raising questions about the optimal surgical technique.

The most common paediatric ACL reconstruction (ACLR) techniques include complete transphyseal, partial transphyseal and physeal‐sparing [16, 37]. Complete transphyseal reconstruction, involving transphyseal tunnels for both femoral and tibial sides, is similar to techniques used for adult patients [1, 16], with some modifications [16, 30, 34]. A previous survey study of this technique reported that if growth disturbances arise, 80% involve the femoral side [19, 29]. To specifically avoid growth deformity in the femur, the partial transphyseal technique with an epiphyseal femoral tunnel and a transphyseal tibial tunnel has been used [6, 16, 19, 37]. To further lower the risk of physeal disturbance in young patients, the physeal‐sparing technique has been developed with both femoral and tibial tunnels in the epiphyses [28, 40]. Finally, a modified Macintosh technique without femoral and tibial tunnel reaming is performed frequently for patients from 8 to 10 years old [37]. This technique combines intra‐articular and extra‐articular reconstruction with the autogenous iliotibial band (IT band) without tunnel drilling [28].

Complete transphyseal, partial transphyseal and physeal‐sparing are all commonly used in the pre‐adolescent/early adolescent age range (11–13 years old) [37]. Previous cadaveric studies have suggested comparable knee stability following various techniques such as physeal‐sparing, partial transphyseal over‐the‐top, and modified Macintosh reconstruction [26], but it is crucial to note that these studies primarily involved adult knee specimens (range: 45–61 years) [35]. There is an increasing appreciation that ACL morphology (length, CSA, coronal and sagittal angle), ACL and bundle function (force and stiffness), as well as joint laxity and stiffness change during skeletal growth [8, 9, 22, 23]. Therefore, it is essential to evaluate paediatric ACLR techniques within age‐specific joints. Due to the extremely limited availability of cadaveric human paediatric knees, there is a lack of data to compare different techniques.

The porcine model has been widely used in musculoskeletal studies due to its similarity in age‐specific biomechanics and anatomy with human knees and the ability to provide a ready supply of paediatric joints at consistent ages [11, 27, 41]. The cross‐sectional area (CSA) and length of porcine ACL increase throughout skeletal growth, while the orientation also changes [9, 23], similar to humans [8, 22]. Biomechanically, joint laxity decreases with age [9, 10, 23], similar to humans during growth [4, 17, 21]. Therefore, the objective of the current study was to investigate and compare the initial joint stability and tissue function following complete transphyseal, partial transphyseal, and physeal‐sparing ACLR in the early adolescent porcine model. To accomplish this, we used a robotic testing system to assess joint biomechanics before and after ACLR using different techniques.

## METHODS

### Specimen preparation

**Figure 1:**
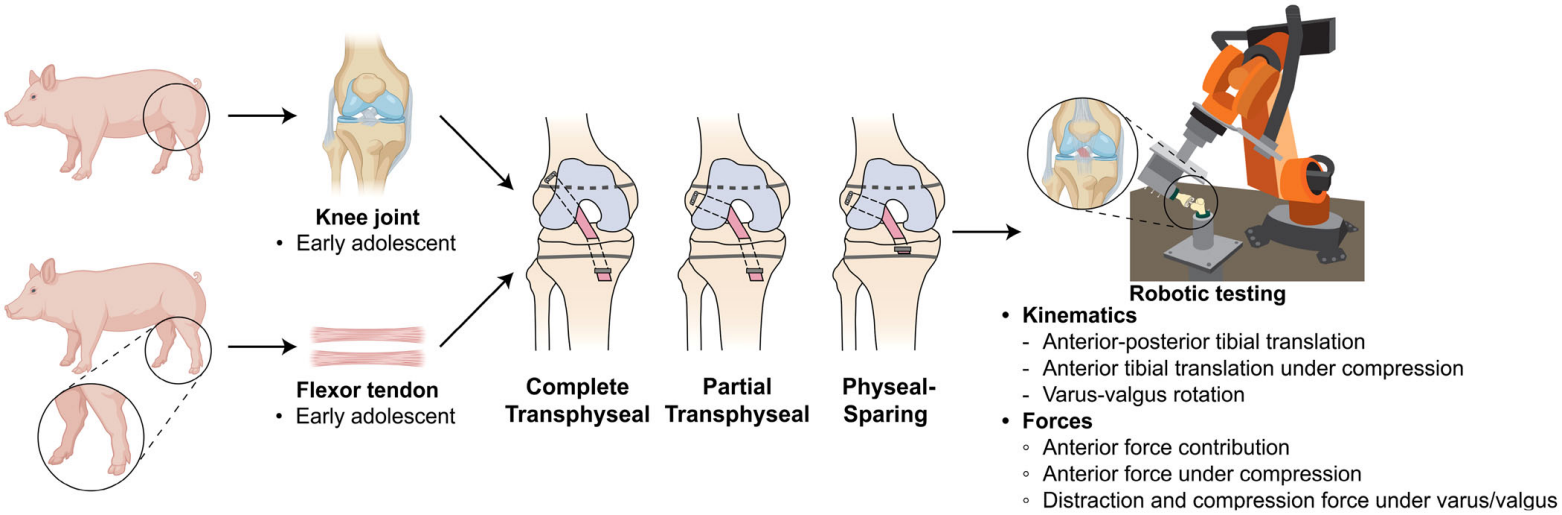
Experimental methods overview. Hindlimbs and flexor tendons were collected from early adolescent (4.5 months of age) pigs. ACL reconstruction (ACLR) was done for each age group using three different paediatric ACLR techniques. A 6‐DOF robotic testing system was utilized to perform biomechanical testing. The joint was tested in intact, ACL‐transection (ACLT), and ACLR states, followed by the removal of other soft tissues. Joint kinematics and soft tissue force contributions were calculated. ACL, anterior cruciate ligament; DOF, degree‐of‐freedom.

A total of 18 deep digital flexor tendons and 18 hindlimbs were collected from female Yorkshire cross‐breed pigs aged 4.5 months (early adolescent) following humane euthanasia. The animals were bred at the North Carolina State University Swine Educational Unit, and the animal use was approved by the North Carolina State University Institutional Animal Use and Care Committee. Our Institutional Animal Care and Use Committee protocol number is #20‐535. Subsequently, the specimens were wrapped in phosphate‐buffered saline (PBS)‐soaked gauze and stored at −20°C. Specimens were divided into three cohorts with different paediatric ACLR techniques: complete transphyseal, partial transphyseal and physeal‐sparing (*n* = 6 legs/technique) (Figure 1). Sample size was based on prior work on paediatric ACLR techniques [26], indicating six samples per group would be sufficient to detect statistically significant differences for effect sizes of 1.8 or greater (80% power, *α* = 0.05).

### Biomechanical testing

A 6‐degree‐of‐freedom robotic testing system (KR300 R2500, KRC4, Kuka) along with a universal force–moment sensor (Omega160 IP65, ATI Industrial Automation) was used for biomechanical testing, as previously described [9, 10, 23]. The system was integrated and controlled using the simVITRO software knee module (Cleveland Clinic). The joints were thawed at room temperature overnight for preparation. Both femur and tibia were fixed within a fibreglass reinforced epoxy compound (Duraglas, USC) in custom aluminium moulds. The prepared joints were wrapped in PBS‐soaked gauze and stored at −20°C again. The flexor tendons and joints were then thawed at room temperature overnight prior to robotic testing.

Each joint was attached to the robotic testing system using custom clamps, with the femur fixed to a platform on the floor and the tibia attached to the end effector of the robot. The coordinate system was defined for each joint using a 3D point digitizer (G2X MicroScribe). During each test, a passive path was initially determined by flexing the joint from 40° of flexion (approximately full extension for porcine knees) to 90° of flexion while minimizing the loads along the remaining five degrees of freedom [23]. Subsequently, anterior‐posterior (AP) forces, compression and varus‐valgus (VV) torques were applied to the tibia at 40° and 60° of flexion since ACL carried higher force closer to full extension rather than deep flexion [33, 47, 48] (Table S1). During each applied loading condition at each flexion angle, internal‐external rotation was also fixed due to the high laxity of the porcine knee in this degree of freedom, allowing adjustment of the remaining three degrees of freedom to maintain forces at 0 N. Two preconditioning trials were conducted for each loading condition. 80 N AP force, 120 N compression and 4 N m VV torque were applied, which were scaled by age groups based on previous studies [9, 23]. For each specimen, the intact joint was first subjected to the loading conditions. The kinematic paths under the applied loads were recorded and then repeated while recording forces for the intact joint. Next, the joint capsule was dissected, followed by repeating kinematics, which carried no more than 1 N anterior force at 40° of flexion. The same loading conditions were then applied after ACL transection (ACLT) while recording the kinematics. The kinematics paths from the intact and ACLT states were repeated for the ACL‐deficient knee while recording forces. Subsequently, ACL reconstruction (ACLR) was performed, and the applied loads were then repeated post‐ACLR, with kinematics recorded and then repeated while recording forces. The recorded ACLR kinematics were repeated after removing the graft. Lastly, to assess the loads taken by other soft tissues, kinematics from the intact, ACLT, and ACLR states were repeated with forces recorded after the sequential removal of the medial collateral ligament (MCL), lateral collateral ligament (LCL), medial meniscus (MMEN), lateral meniscus (LMEN), medial femoral condyle and lateral femoral condyle.

AP tibial translation (APTT) under applied AP force, anterior tibial translation (ATT) under applied compression, and VV rotation under applied VV torques were obtained when applying loading conditions. Delta APTT, delta ATT under compression, and delta VV rotation relative to the intact state were then calculated for the ACLT and ACLR states. The anterior forces taken by the native ACL and reconstructed ACL graft under maximum anterior translation, compression, varus rotation, and valgus rotation were calculated using the principle of superposition [13, 18, 23]. The anterior forces within the MCL were calculated under maximum anterior translation and compression. The distraction forces taken by the collateral ligaments and the compression forces taken by the menisci were calculated under maximum varus and valgus rotation. The resultant forces of the ACL, graft, collateral ligaments and menisci were calculated under varus and valgus rotation. For all tissue forces, the absolute delta forces were calculated relative to the intact state.

### Surgical procedure

All reconstructions were performed by a single surgeon via an arthrotomy. Flexor tendons harvested from early adolescent pigs were used as ACL grafts. The diameter of the femoral and tibial tunnels was determined as 8 mm based on prior data on porcine ACL size across skeletal growth [23]. The graft size was standardized using an 8‐mm‐diameter sizer. Complete transphyseal reconstruction involved the creation of transphyseal tunnels on both the femoral and tibial sides. In contrast, the partial transphyseal technique featured a transphyseal tunnel on the tibial side and an epiphyseal tunnel on the femoral side. The physeal‐sparing technique utilized epiphyseal tunnels for both the femoral and tibial tunnels (Figure 1). For each technique, a guide wire was inserted into the midpoint between the anteromedial (AM) and posterolateral (PL) bundle footprints on the tibia using an ACL tip aimer system at 55° (Smith & Nephew). A cannulated reamer (8 mm diameter, Arthrex) was then used to overream the tunnel. Femoral tunnel placement was achieved by inserting a guide pin into the centre of the AM and PL bundle footprints with AM drilling, followed by overdrilling with a reamer. The graft was then passed through the tibial and femoral tunnels. Femoral fixation was performed by a #2 FiberWire (Arthrex) with an ABS button (Arthrex). Next, the knee was preconditioned by five cycles of passive flexion‐extension while applying a 22 N pretension to the tibial side of graft. Finally, the tibia side was fixed by two staples (Arthrex) with 100 N graft pretension. Tension was applied and monitored using a digital force gauge. Tibial fixation was performed at maximum posterior translation at 40° of knee flexion. Following completion of all tests, validation of tunnel position was performed by sectioning the bones along the tunnels to confirm their positions relative to the femoral and tibial physes.

### Statistics

Statistical analysis was conducted using Prism (GraphPad). Within each technique, kinematics data from intact, ACLT and ACLR states were compared using one‐way ANOVA followed by Tukey's honestly significant difference (HSD) post hoc test. Changes in joint kinematics and tissue forces relative to the intact joint were compared across different techniques using one‐way ANOVA followed by Tukey's HSD post hoc test. A paired *t* test was performed to compare soft tissue forces before and after ACLR by each surgical technique. In all analyses, statistical significance was defined as *p* < 0.05. Individual data points, along with mean values, were shown in the figures. Mean differences, 95% confidence intervals (CIs) of differences, and *p* values for all statistics are included as Tables S2–S35.

## RESULTS

**Figure 2:**
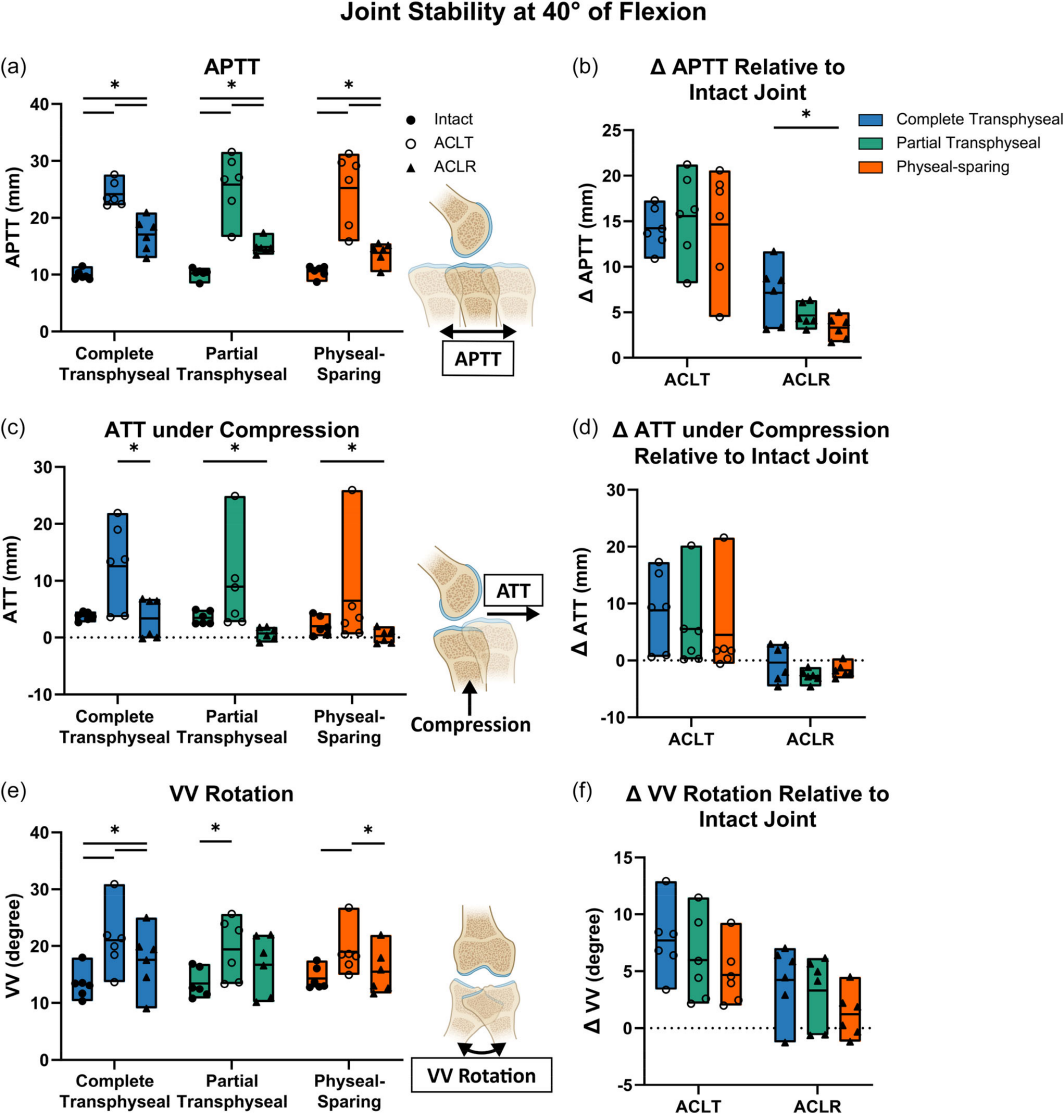
Physeal‐sparing technique showed better ability to restore anterior joint stability in early adolescent porcine joints at 40° of flexion. (a) All techniques could not restore anterior‐posterior tibial translation (APTT) to intact state. (b) Increases in APTT following physeal‐sparing technique were significantly smaller than those after complete transphyseal technique. (c) Anterior tibial translation (ATT) under compression following ACLR was smaller than intact state only when using partial transphyseal and physeal‐sparing technique. (d) Complete transphyseal led to greater increases in ATT under compression relative to intact state. (e) Varus‐valgus (VV) rotation was restored to intact state when using partial transphyseal and physeal‐sparing technique but not the complete transphyseal technique. (f) VV increases relative to intact state were variable across techniques. Data points presented with mean values as bars. Statistical significance (*p* < 0.05) between states indicated (*).

Joint stability was quantitatively evaluated under the same loading conditions under intact, ACLT and ACLR states to compare reconstruction techniques (Table S2–S16). Under AP drawer at 40° of flexion, anterior joint stability was assessed using APTT (Figure 2a) and the change in APTT relative to the intact state (Figure 2b). ACLT resulted in an average increase in APTT of 14.2–15.6 mm across all techniques relative to the intact state, all of which were found to be statistically significant. ACLR partially restored anterior stability but did not fully return to the intact state. The average increase in APTT was 7.1 mm for complete transphyseal, 4.7 mm for partial transphyseal and 3.3 mm for physeal‐sparing ACLR, with all increases being statistically significant (Figure 2a). Between techniques, the average increase of APTT significantly differed by 3.8 mm between complete transphyseal and physeal‐sparing technique (95% CI: [0.6–7.1], *p* = 0.02) (Figure 2b). Under compression forces, ATT increased by an average of 4.5–8.8 mm after ACLT (Figure 2c). Following reconstruction, the average ATT was 2.8 mm smaller than the intact state following partial transphyseal ACLR (95% CI: [1.3–4.3], *p* = 0.004) and 1.7 mm following physeal‐sparing ACLR (95% CI: [0.1–3.4], *p* = 0.04), with no significant difference in the average ATT changes across techniques (Figure 2d). Under VV torque, VV rotation significantly increased after ACLT and was restored to the intact state after partial transphyseal and physeal‐sparing techniques (Figure 2e). The average increase of VV rotation following physeal‐sparing ACLR (1.2°) was smaller on average relative to complete transphyseal (4.2°) and partial transphyseal reconstruction (3.3°), but no statistically significant differences across techniques were found (Figure 2f, Table S16).

**Figure 3:**
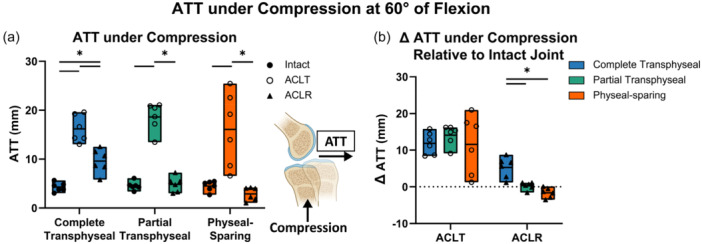
Partial transphyseal and physeal‐sparing showed better anterior stability under compression compared to complete transphyseal technique at 60° of flexion. (a) Anterior tibial translation (ATT) under compression following ACLR was comparable to intact state only when using partial transphyseal and physeal‐sparing technique. (b) Complete transphyseal led to greater increases in ATT under compression relative to intact state. Data points presented with mean values as bars. Statistical significance (*p* < 0.05) between states indicated (*).

At 60° of flexion, AP and VV stability were similar to 40° (Figure S1). Under compression forces at 60° of flexion (Tables S17–S21), ATT increased by an average of more than 10 mm after ACLT (Figure 3a). Following reconstruction, the average ATT was restored to within 0.3 mm for partial transphyseal ACLR and decreased by an average of 1.6 mm relative to the intact joint after physeal‐sparing ACLR (Figure 3a), which were 5.0 and 6.9 mm lower than the average increase observed after complete transphyseal ACLR, respectively (*p* ≤ 0.001 for each) (Figure 3b, Table S21). The differences in joint stability between ACLR and ACLT states were also assessed (Figure S2).

**Figure 4:**
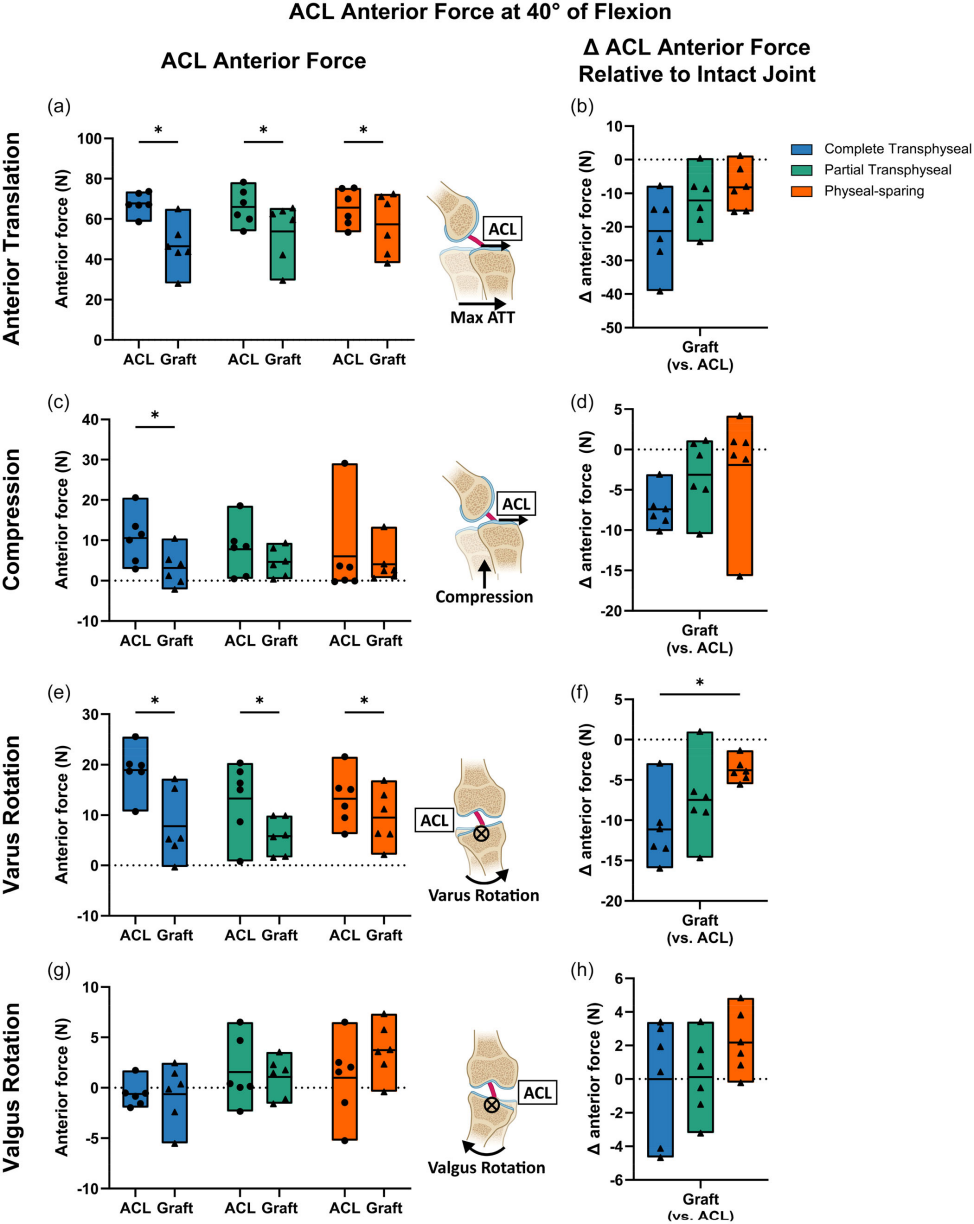
Anterior forces taken by ACL decreased following ACL reconstruction (ACLR) at 40° of flexion. (a) All techniques could not restore reconstructed ACL graft anterior force under anterior translation to native ACL, (b) with no difference in the changes between techniques. (c) Anterior forces taken by reconstructed ACL graft were not comparable to native ACL under compression following complete transphyseal ACLR. (d) Changes in ACL anterior force were similar across techniques. (e) Graft anterior force under varus rotation decreased following ACLR. (f) The decrease in ACL anterior force was smaller following physeal‐sparing technique compared to complete transphyseal. (g) Anterior force under valgus rotation was variable, (h) with no difference between techniques. ⊗ represents the ACL anterior force direction pointing into boards. Data points presented with mean values as bars. Statistical significance (*p* < 0.05) between states indicated (*). ACL, anterior cruciate ligament.

Under each joint position, the tissue function of the native ACL and reconstructed ACL graft was evaluated by the anterior force and the change in anterior force relative to the intact state (Tables S22–S29). In all intact specimens at 40° of flexion, the native ACL predominantly carried the applied anterior force under maximum anterior translation (~80% on average), while the force on the graft significantly decreased relative to the intact ACL (Figure 4a). Between techniques, there was no significant difference in the average decrease of graft anterior force compared to the native ACL across different techniques (Figure 4b, Table S23). Similarly, the anterior force taken by the graft was significantly smaller than the native ACL under maximum compression following complete transphyseal ACLR (−7 N, 95% CI: [−10, 5], *p* < 0.001), but not following partial transphyseal and physeal‐sparing ACLR (Figure 4c). There was no difference in the changes across different techniques (main effect *p* = 0.54) (Figure 4d). The graft anterior force under varus rotation decreased following all techniques (Figure 4e). The decrease following physeal‐sparing ACLR was 7 N smaller than that after complete transphyseal ACLR (95% CI: [−13, −1], *p* = 0.02) (Figure 4f). Under maximum valgus rotation, ACL anterior forces were smaller than 10 N both before and after ACLR (Figure 4g), with no difference across techniques (main effect *p* = 0.36) (Figure 4h). Similar results in ACL anterior force were found at 60° of flexion (Figure S3).

**Figure 5:**
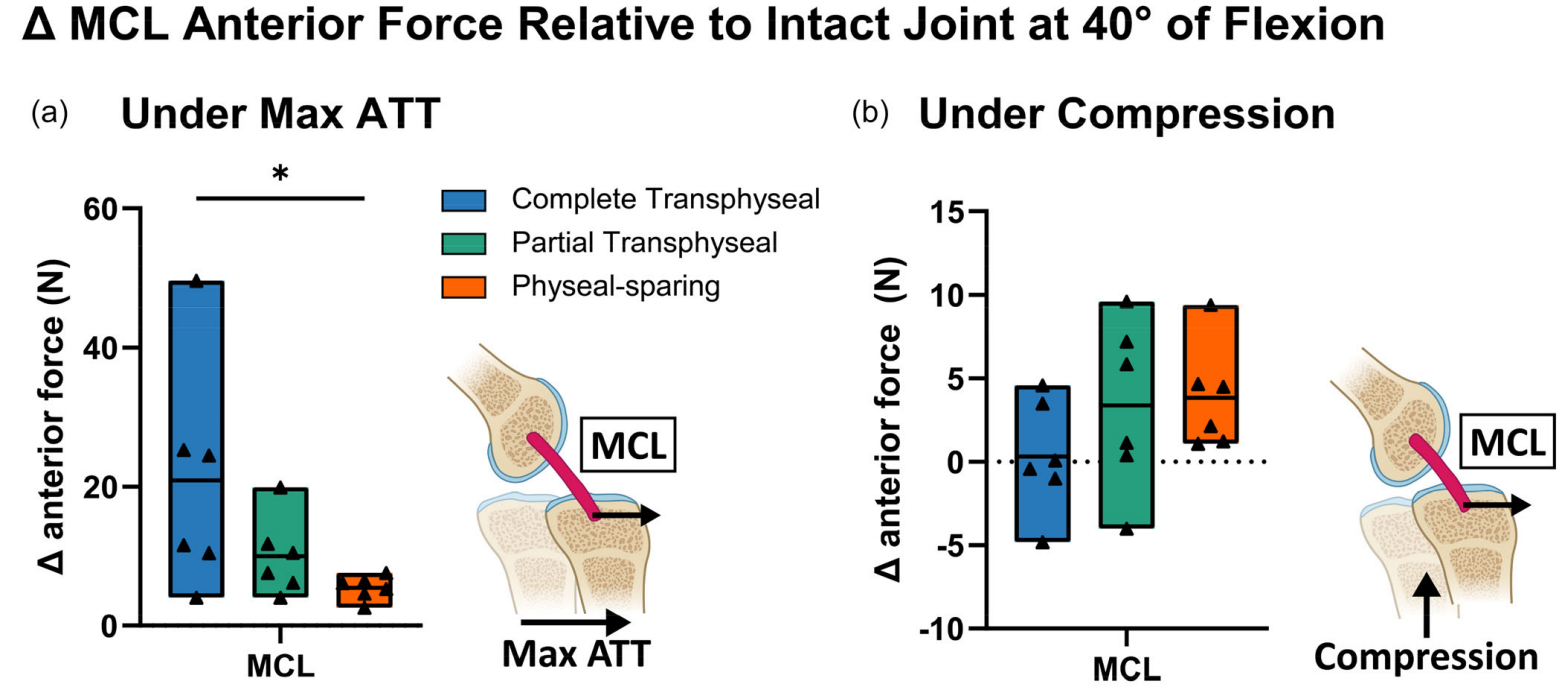
Increases in MCL anterior force were higher following complete transphyseal ACLR at 40° of flexion. (a) Anterior forces under anterior translation taken by MCL increased following ACLR by different techniques. (b) More anterior forces under anterior translation shifted to MCL following complete transphyseal technique, while changes in MCL anterior forces were similar across techniques under compression. Data points presented with mean values as bars. Statistical significance (*p* < 0.05) between techniques indicated (*). ACLR, anterior cruciate ligament reconstruction; MCL, medial collateral ligament.

MCL is a secondary stabilizer to resist the ATT when ACL is injured. Therefore, the anterior force taken by MCL was also quantified by the anterior force and the change in anterior force relative to the intact state at 40° of flexion (Tables S30 and S31). Under maximum anterior translation, the anterior force carried by MCL increased by 21 N, 10 N and 5 N following complete transphyseal, partial transphyseal and physeal‐sparing ACLR, respectively (Figure 5a). When performing ACLR using the complete transphyseal technique, the average increase of the MCL anterior force was 16 N higher than physeal‐sparing techniques (95% CI: [0, 31], *p* = 0.04) (Figure 5a). Under maximum compression, the increases of MCL anterior forces were relatively small, with no differences in the changes across different techniques (main effect *p* = 0.25) (Figure 5b). Similar results in MCL anterior force were found at 60° of flexion (Figure S4).

**Figure 6:**
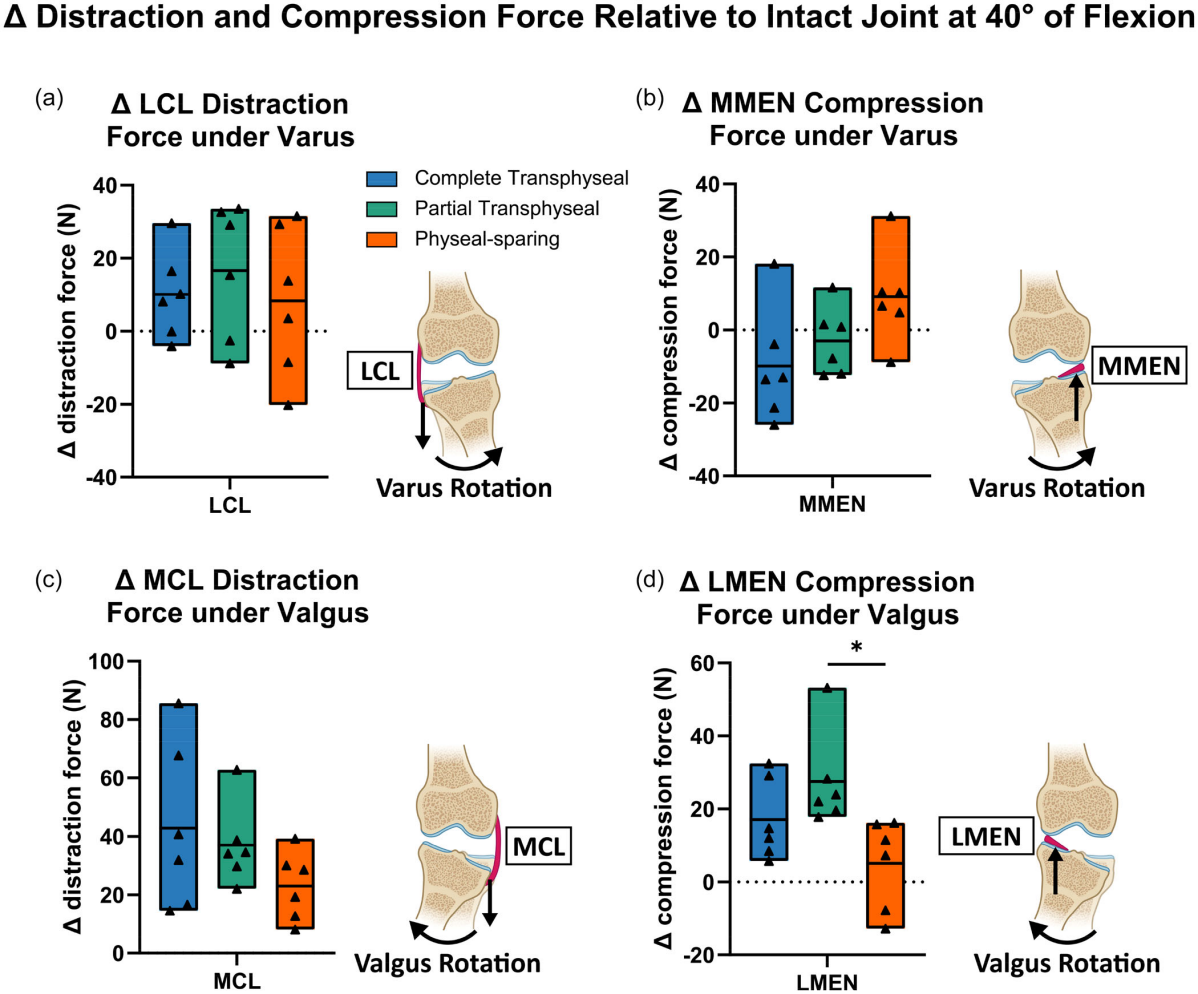
Distraction forces taken by collateral ligaments as well as compression forces taken by menisci were assessed under maximum varus‐valgus rotation at 40° of flexion. (a) Increases in LCL distraction forces under varus rotation were similar across techniques. (b) Compression forces on the medial meniscus (MMEN) under varus rotation showed variability, with no differences in changes following ACL reconstruction (ACLR) by different techniques. (c) No differences were observed in increases in MCL distraction forces under valgus rotation across techniques. (d) Compression forces on the lateral meniscus (LMEN) under valgus rotation increased following partial transphyseal ACLR but decreased following physeal‐sparing ACLR. Data points presented with mean values as bars. Statistical significance (*p* < 0.05) between techniques indicated (*). ACL, anterior cruciate ligament; LCL, lateral collateral ligament; MCL, medial collateral ligament.

Force along the distraction‐compression direction taken by collateral ligaments and menisci were investigated at 40° of flexion (Tables S32–S35). Under maximum varus rotation, there was no difference in the average increase of LCL distraction forces across different techniques (main effect *p* = 0.31) (Figure 6a). Similarly, the changes of medial meniscus compression forces were similar across techniques (main effect *p* = 0.86) (Figure 6b). Under maximum valgus rotation, MCL distraction forces increased on average by 43 N following complete transphyseal, 37 N following partial transphyseal and 23 N following physeal‐sparing ACLR (Figure 6c). There was no significant difference in these increases across techniques (Figure 6c, Table S34). The average increase of compression forces in the lateral meniscus following partial transphyseal ACLR was 22 N greater than the changes in compression forces after physeal‐sparing ACLR (95% CI: [4, 40], *p* = 0.02) (Figure 6d). Similar results were observed at 60° of flexion (Figure S5).

## DISCUSSION

The aim of this study was to assess various paediatric ACLR techniques through biomechanical analysis using age‐specific grafts and porcine joints. Physeal‐sparing techniques revealed a superior ability to restore anterior knee stability compared to the complete transphyseal technique in early adolescent joints. Joints following partial transphyseal and physeal‐sparing ACLR achieved similar levels of joint stability under joint compression and VV rotation, although neither technique fully restored the AP joint stability under anterior forces alone. The anterior forces under anterior tibial loading shifted to the MCL more for the complete transphyseal technique compared to the physeal‐sparing technique. Under valgus torque, the change in compression force of the lateral meniscus was smaller in the physeal‐sparing technique compared to the partial transphyseal technique.

There is high variability in the selection of surgical techniques for treating paediatric patients aged 11–13 years [37]. Notably, the choice of paediatric ACLR technique varies greatly among surgeons and is significantly associated with the surgeon's fellowship training rather than the joint and ACL function [37]. The most important implication of our current study is to provide biomechanical insights for technique selection when treating paediatric ACL injuries. Joint laxity has been found to be a risk factor associated with ACL injury in adults [42, 45], which also decreases during skeletal growth in paediatric populations [8, 23, 43, 44]. Therefore, evaluating how different techniques affect initial joint stability at various skeletal development stages can enhance understanding of paediatric ACLR techniques. An early adolescent porcine model was used in this work rather than adult human specimens, which were commonly used in previous studies. Although full extension for porcine joints is approximately 40°, and thus different from human joints, the morphological characteristics (CSA, length and orientation) of the paediatric porcine ACL are similar to those of paediatric human ACL, making it a more anatomically relevant model [8, 23]. Adult human specimens with a lack of open physis could not replicate the joint stability and ACL morphology of a paediatric knee due to their higher joint stiffness, lower joint laxity and different morphology. In the current study, partial transphyseal and physeal‐sparing techniques showed a better ability to restore initial anterior stability under anterior forces and compression compared to the complete transphyseal technique in early adolescent joints. The evaluation of anterior stability under compression and VV stability in our study was rarely addressed in previous work. The variation in joint stability following different techniques is notable and should be a consideration when choosing surgical approaches for patients at different ages.

Femoral tunnel orientation could be a factor in the differences in joint stability and tissue forces between techniques since the partial transphyseal and physeal‐sparing techniques used a sharper angle to avoid crossing the physis [36]. Previous biomechanical work with skeletally mature human cadaveric joints indicated that the 10 o'clock femoral tunnel position showed better rotatory stability at 15° and 30° of flexion when compared to the 11 o'clock position [32]. Another similar human study demonstrated that a less vertical femoral tunnel placement led to smaller ATT as well as higher graft loads at 30° of flexion [3]. Others suggested that femoral tunnel position is important for paediatric ACLR techniques [36]. Tibial tunnel orientation in the physeal‐sparing technique was also less vertical in the coronal and sagittal planes compared to the transphyseal tunnels [26]. A prior computational model indicated that tibial tunnel position was also a key factor in knee laxity [38], namely that a more horizontal orientation of the tibial tunnel would lead to larger AP joint laxity [26, 38]. In contrast, in the current study, the partial transphyseal and physeal‐sparing techniques showed similar joint stability despite the more vertical tibial tunnel orientation in the partial transphyseal technique compared to the physeal‐sparing technique.

The kinematic data in the current study are largely consistent with previous research. The large joint destabilization following ACLT is consistent with previous studies using similarly aged porcine joints [10, 23]. Following ACLR, the AP kinematics under AP load showed an average increase of APTT ranging from 3.3 to 7.1 mm compared to the intact state at 40° of flexion. These findings were comparable to prior work, which applied an 89 N anterior tibial force to skeletally mature porcine joints after complete transphyseal ACLR and found a differential laxity ranging from 3.7 to 9.6 mm at 30° [12, 24]. One study using 7‐ to 8‐month‐old porcine joints showed an increased ATT within 2 mm at 30° under 89 N anterior tibial load [25]. The minor differences in the joint kinematics may be due to the ACLR, in that study was performed arthroscopically, but it was an open surgery in our work. Additionally, the previous study did not measure the posterior laxity of the joint, so the full range of APTT cannot be compared. It is possible that the tibia in the ‘zero’ position may have already been more anterior than normal, thus obscuring excessive laxity. In human joints, the physeal‐sparing technique could restore the increase of APTT within 2 mm of the intact state under a 100 N anterior load [26]. However, the specimens from that study were all adult knee joints (mean age: 62.3 years) due to the difficulty of obtaining skeletally immature human joints, and the complete transphyseal technique was not performed [26]. In terms of comparison between techniques, the current results for anterior stability align with a previous human paediatric study including patients with a mean age closer to adolescence (mean age: 13.1 years) [36], which found that the physeal‐sparing technique resulted in better post‐operative joint laxity under Lachman and pivot‐shift tests. However, the changes in laxity between techniques in this human work were practically similar (complete transphyseal: 2.0 mm, partial transphyseal: 1.7 mm and physeal‐sparing: 0.2 mm) [36]. The VV stability in our study was comparable to a human study that applied a 5 N m varus torque, demonstrating similar rotational stability between the intact joint and the joint following physeal‐sparing ACLR [26].

A major function of the native ACL is to resist anterior tibial loading, which makes the load carried by the graft important to maintain joint stability following ACLR. MCL was the secondary stabilizer against the anterior translation in this study. The average increase of the anterior MCL force was higher following complete transphyseal ACLR compared to physeal‐sparing ACLR. The compression force taken by the lateral meniscus under valgus torque increased more following partial transphyseal ACLR compared to physeal‐sparing ACLR. These findings indicated that the joint reconstructed with the physeal‐sparing technique showed smaller changes in the overall tissue forces compared to the transphyseal techniques, which may limit the risk of secondary injury or long‐term degeneration.

This study has a few limitations. First, although statistical differences were achieved between surgical techniques, a larger sample size might reveal additional statistical differences in kinematics and forces. Additionally, although the current study used a paediatric porcine model, the range of flexion angle of porcine knee joints is smaller than that of humans, with full extension occurring at approximately 40°. Evaluation at or near full extension in humans (0°) might provide further insights into joint function between techniques. Moreover, this study presents a time‐zero analysis of initial joint biomechanics following ACLR and does not consider other factors such as graft remodelling and active muscle forces. Future in vivo studies comparing long‐term degenerative outcomes across different paediatric ACLR techniques using age‐specific models would be beneficial. The role of the lateral extra‐articular tendons (LET) was not assessed in this study, but it showed an enhanced joint stability when combined with ACLR in paediatric patients [5, 39]. Finally, the IT band is not clearly defined within porcine hindlimbs, preventing the inclusion of the modified Macintosh procedure and the partial transphyseal over‐the‐top technique in this study.

In conclusion, this study compared complete transphyseal, partial transphyseal, and physeal‐sparing reconstruction techniques in a porcine model of early adolescent joints. The physeal‐sparing technique showed better ability to restore anterior stability compared to the complete transphyseal technique, with less transfer of anterior force from the reconstructed ACL graft to the MCL. This potentially ensures better long‐term joint stability and higher activity levels. This study highlights the importance of evaluating different paediatric ACLR techniques using age‐specific knee joints, contributing to a deeper understanding of surgical interventions for ACL injuries in young patients.

## AUTHOR CONTRIBUTIONS

Yukun Zhang contributed to the development of research design, acquisition, analysis, interpretation of data and drafting of the manuscript. Kaan Gurbuz contributed to the development of research design, acquisition, analysis, interpretation of data and revision of the manuscript. Logan Opperman and Jeffrey T. Spang contributed to the development of research design, interpretation of data and revision of the manuscript. Matthew B. Fisher contributed to the development of research design, acquisition and interpretation of data, and revision of the manuscript. All authors have read and approved the final submitted manuscript.

## CONFLICT OF INTEREST STATEMENT

The authors declare no conflicts of interest.

## ETHICS STATEMENT

The animal use was approved by the North Carolina State University Institutional Animal Use and Care Committee. Our Institutional Animal Care and Use Committee protocol number is #20‐535.

## Supporting information

Supporting information.

## Data Availability

The data that support the findings of this study are available from the corresponding author upon request.

## References

[1] Anderson AF . Transepiphyseal replacement of the anterior cruciate ligament in skeletally immature patients: a preliminary report. J Bone Joint Surg Am. 2003;85(7):1255–1263. 12851350 10.2106/00004623-200307000-00011

[2] Anderson AF , Anderson CN . Correlation of meniscal and articular cartilage injuries in children and adolescents with timing of anterior cruciate ligament reconstruction. Am J Sports Med. 2015;43(2):275–281. 25497145 10.1177/0363546514559912

[3] Araujo PH , Asai S , Pinto M , Protta T , Middleton K , Linde‐Rosen M , et al. ACL graft position affects in situ graft force following ACL reconstruction. J Bone Jt Surg. 2015;97(21):1767–1773. 10.2106/JBJS.N.00539 26537164

[4] Baxter MP . Assessment of normal pediatric knee ligament laxity using the genucom. J Pediatr Orthop. 1988;8(5):546–550. 3170733 10.1097/01241398-198809000-00010

[5] Carrozzo A , Monaco E , Saithna A , Annibaldi A , Guy S , Ferreira A , et al. Clinical outcomes of combined anterior cruciate ligament reconstruction and lateral extra‐articular tenodesis procedures in skeletally immature patients: a systematic review from the SANTI study group. J Pediatr Orthop. 2023;43(1):24–30. 35980761 10.1097/BPO.0000000000002236

[6] Chambers CC , Monroe EJ , Allen CR , Pandya NK . Partial transphyseal anterior cruciate ligament reconstruction: clinical, functional, and radiographic outcomes. Am J Sports Med. 2019;47(6):1353–1360. 30995077 10.1177/0363546519836423

[7] Collins MJ , Arns TA , Leroux T , Black A , Mascarenhas R , Bach Jr, BR , et al. Growth abnormalities following anterior cruciate ligament reconstruction in the skeletally immature patient: a systematic review. Arthroscopy. 2016;32(8):1714–1723. 27161510 10.1016/j.arthro.2016.02.025

[8] Cone SG , Barnes RH , Howe D , Fordham LA , Fisher MB , Spang JT . Age‐ and sex‐specific differences in ACL and ACL bundle size during adolescent growth. J Orthop Res. 2022;40(7):1613–1620. 34727387 10.1002/jor.25198 PMC9058042

[9] Cone SG , Lambeth EP , Ru H , Fordham LA , Piedrahita JA , Spang JT , et al. Biomechanical function and size of the anteromedial and posterolateral bundles of the ACL change differently with skeletal growth in the pig model. Clin Orthop Relat Res. 2019;477(9):2161–2174. 31373947 10.1097/CORR.0000000000000884. PMC7000103

[10] Cone SG , Piedrahita JA , Spang JT , Fisher MB . In situ joint stiffness increases during skeletal growth but decreases following partial and complete anterior cruciate ligament injury. J Biomech Eng. 2019;141(12):121001. 31513698 10.1115/1.4044582 PMC7105148

[11] Cone SG , Warren PB , Fisher MB . Rise of the pigs: utilization of the porcine model to study musculoskeletal biomechanics and tissue engineering during skeletal growth. Tissue Eng Part C Methods. 2017;23(11):763–780. 28726574 10.1089/ten.tec.2017.0227 PMC5689129

[12] Debandi A , Maeyama A , Lu S , Hume C , Asai S , Goto B , et al. Biomechanical comparison of three anatomic ACL reconstructions in a porcine model. Knee Surg Sports Traumatol Arthrosc. 2011;19(5):728–735. 21153539 10.1007/s00167-010-1338-3

[13] Debski RE , Yamakawa S , Musahl V , Fujie H . Use of robotic manipulators to study diarthrodial joint function. J Biomech Eng. 2017;139(2):021010. 10.1115/1.4035644 28056127

[14] Dodwell ER , LaMont LE , Green DW , Pan TJ , Marx RG , Lyman S . 20 years of pediatric anterior cruciate ligament reconstruction in New York state. Am J Sports Med. 2014;42(3):675–680. 24477820 10.1177/0363546513518412

[15] Dumont GD , Hogue GD , Padalecki JR , Okoro N , Wilson PL . Meniscal and chondral injuries associated with pediatric anterior cruciate ligament tears: relationship of treatment time and patient‐specific factors. Am J Sports Med. 2012;40(9):2128–2133. 22729621 10.1177/0363546512449994

[16] Ellis HB , Zak TK , Jamnik A , Lind DRG , Dabis J , Losito M , et al. Management of pediatric anterior cruciate ligament injuries: a critical analysis. JBJS Reviews. 2023;11(8):e22. 10.2106/JBJS.RVW.22.00223 37535763

[17] Flynn JM , Mackenzie W , Kolstad K , Sandifer E , Jawad AF , Galinat B . Objective evaluation of knee laxity in children. J Pediatr Orthop. 2000;20(2):259–263. 10739294

[18] Fujie H , Livesay GA , Woo SL‐Y , Kashiwaguchi S , Blomstrom G . The use of a universal force‐moment sensor to determine in‐situ forces in ligaments: a new methodology. J Biomech Eng. 1995;117(1):1–7. 7609472 10.1115/1.2792266

[19] Gamble JG , Shirodkar RN , Gamble JG . Knee valgus and patellofemoral instability after pediatric anterior cruciate ligament reconstruction: a case report and review of the literature. J Med Case Rep. 2023;17(1):212. 37211594 10.1186/s13256-023-03920-2 PMC10201725

[20] Gracia G , Thévenin‐Lemoine C , Laumonerie P , Sales de Gauzy J , Accadbled F , French Arthroscopy S . Anterior cruciate ligament tears in children: management and growth disturbances. A survey of French Arthroscopy Society members. Orthop Traumatol Surg Res. 2019;105(4):747–750. 30982775 10.1016/j.otsr.2019.02.017

[21] Hinton RY , Rivera VR , Pautz MJ , Sponseller PD . Ligamentous laxity of the knee during childhood and adolescence. J Pediatr Orthop. 2008;28(2):184–187. 18388713 10.1097/BPO.0b013e3181652120

[22] Hosseinzadeh S , Kiapour AM . Age‐related changes in ACL morphology during skeletal growth and maturation are different between females and males. J Orthop Res. 2021;39(4):841–849. 32427346 10.1002/jor.24748 PMC7674212

[23] Howe D , Cone SG , Piedrahita JA , Collins B , Fordham LA , Griffith EH , et al. Sex‐specific biomechanics and morphology of the anterior cruciate ligament during skeletal growth in a porcine model. J Orthop Res. 2022;40(8):1853–1864. 34751996 10.1002/jor.25207 PMC9081289

[24] Iriuchishima T , Tajima G , Ingham SJM , Shen W , Horaguchi T , Saito A , et al. Intercondylar roof impingement pressure after anterior cruciate ligament reconstruction in a porcine model. Knee Surg Sports Traumatol Arthrosc. 2009;17(6):590–594. 19089408 10.1007/s00167-008-0691-y

[25] Kato Y , Ingham SJM , Kramer S , Smolinski P , Saito A , Fu FH . Effect of tunnel position for anatomic single‐bundle ACL reconstruction on knee biomechanics in a porcine model. Knee Surg Sports Traumatol Arthrosc. 2010;18(1):2–10. 19784631 10.1007/s00167-009-0916-8

[26] Kennedy A , Coughlin DG , Metzger MF , Tang R , Pearle AD , Lotz JC , et al. Biomechanical evaluation of pediatric anterior cruciate ligament reconstruction techniques. Am J Sports Med. 2011;39(5):964–971. 21257848 10.1177/0363546510390189

[27] Kiapour AM , Shalvoy MR , Murray MM , Fleming BC . Validation of porcine knee as a sex‐specific model to study human anterior cruciate ligament disorders. Clin Orthop Relat Res. 2015;473(2):639–650. 25269532 10.1007/s11999-014-3974-2 PMC4294889

[28] Kocher MS , Garg S , Micheli LJ . Physeal sparing reconstruction of the anterior cruciate ligament in skeletally immature prepubescent children and adolescents: surgical technique. J Bone Jt Surg. 2006;88(1):283–293. 10.2106/JBJS.F.00441 16951100

[29] Kocher MS , Saxon HS , Hovis WD , Hawkins RJ . Management and complications of anterior cruciate ligament injuries in skeletally immature patients: survey of the herodicus society and the ACL study group. J Pediatr Orthop. 2002;22(4):452–457. 12131440

[30] Kocher MS , Smith JT , Zoric BJ , Lee B , Micheli LJ . Transphyseal anterior cruciate ligament reconstruction in skeletally immature pubescent adolescents. J Bone Joint Surg Am. 2007;89(12):2632–2639. 18056495 10.2106/JBJS.F.01560

[31] Kooy CEW , Jakobsen RB , Fenstad AM , Persson A , Visnes H , Engebretsen L , et al. Major increase in incidence of pediatric ACL reconstructions from 2005 to 2021: a study from the Norwegian Knee Ligament Register. Am J Sports Med. 2023;51(11):2891–2899. 37497771 10.1177/03635465231185742 PMC10478322

[32] Loh JC , Fukuda Y , Tsuda E , Steadman RJ , Fu FH , Woo SL . Knee stability and graft function following anterior cruciate ligament reconstruction: comparison between 11 o'clock and 10 o'clock femoral tunnel placement. 2002 Richard O'Connor Award paper. Arthroscopy. 2003;19(3):297–304. 12627155 10.1053/jars.2003.50084

[33] Marieswaran M , Jain I , Garg B , Sharma V , Kalyanasundaram D . A review on biomechanics of anterior cruciate ligament and materials for reconstruction. Appl Bionics Biomech. 2018;2018(1):4657824. 29861784 10.1155/2018/4657824 PMC5971278

[34] Mathew S , Ellis HB , Wyatt CW , Sabatino MJ , Zynda AJ , Dennis G , et al. Is anteromedial drilling safe in transphyseal anterior cruciate ligament reconstruction in adolescents with growth remaining? J Pediatr Orthop. 2019;39(4):e278–e283. 30702639 10.1097/BPO.0000000000001289

[35] McCarthy MM , Tucker S , Nguyen JT , Green DW , Imhauser CW , Cordasco FA . Contact stress and kinematic analysis of all‐epiphyseal and over‐the‐top pediatric reconstruction techniques for the anterior cruciate ligament. Am J Sports Med. 2013;41(6):1330–1339. 23613444 10.1177/0363546513483269 PMC4041132

[36] Pagliazzi G , Cuzzolin M , Pacchiarini L , Delcogliano M , Filardo G , Candrian C . Physeal‐sparing ACL reconstruction provides better knee laxity restoration but similar clinical outcomes to partial transphyseal and complete transphyseal approaches in the pediatric population: a systematic review and meta‐analysis. Knee Surg Sports Traumatol Arthrosc. 2023;31(1):206–218. 35838794 10.1007/s00167-022-07032-0

[37] Patel NM , Talathi NS , Talwar D , Fabricant PD , Kocher MS , Ganley TJ , et al. Factors affecting the preferred surgical technique in pediatric anterior cruciate ligament reconstruction. Orthop J Sports Med. 2018;6(9):2325967118796171. 30246042 10.1177/2325967118796171 PMC6146329

[38] Peña E , Calvo B , Martinez MA , Palanca D , Doblaré M . Influence of the tunnel angle in ACL reconstructions on the biomechanics of the knee joint. Clin Biomech. 2006;21(5):508–516. 10.1016/j.clinbiomech.2005.12.013 16472892

[39] Perelli S , Costa GG , Terron VM , Formagnana M , Bait C , Espregueira‐Mendes J , et al. Combined anterior cruciate ligament reconstruction and modified Lemaire lateral extra‐articular tenodesis better restores knee stability and reduces failure rates than isolated anterior cruciate ligament reconstruction in skeletally immature patients. Am J Sports Med. 2022;50(14):3778–3785. 36345894 10.1177/03635465221128926

[40] Pierce TP , Issa K , Festa A , Scillia AJ , McInerney VK . Pediatric anterior cruciate ligament reconstruction: a systematic review of transphyseal versus physeal‐sparing techniques. Am J Sports Med. 2017;45(2):488–494. 27045088 10.1177/0363546516638079

[41] Proffen BL , McElfresh M , Fleming BC , Murray MM . A comparative anatomical study of the human knee and six animal species. Knee. 2012;19(4):493–499. 21852139 10.1016/j.knee.2011.07.005 PMC3236814

[42] Ramesh R , Von Arx O , Azzopardi T , Schranz PJ . The risk of anterior cruciate ligament rupture with generalised joint laxity. J Bone Joint Surg Br. 2005;87–B(6):800–803. 10.1302/0301-620X.87B6.15833 15911662

[43] Shultz SJ , Cruz MR , Casey E , Dompier TP , Ford KR , Pietrosimone B , et al. Sex‐specific changes in physical risk factors for anterior cruciate ligament injury by chronological age and stages of growth and maturation from 8 to 18 years of age. J Athl Train. 2022;57(9–10):830–876. 36638346 10.4085/1062-6050-0038.22 PMC9842121

[44] Shultz SJ , Nguyen A‐D , Schmitz RJ . Differences in lower extremity anatomical and postural characteristics in males and females between maturation groups. J Orthop Sports Phys Ther. 2008;38(3):137–149. 18383647 10.2519/jospt.2008.2645

[45] Uhorchak JM , Scoville CR , Williams GN , Arciero RA , Pierre PSt , Taylor DC . Risk factors associated with noncontact injury of the anterior cruciate ligament. Am J Sports Med. 2003;31(6):831–842. 14623646 10.1177/03635465030310061801

[46] Wong SE , Feeley BT , Pandya NK . Complications after pediatric ACL reconstruction: a meta‐analysis. J Pediatr Orthop. 2019;39(8):e566–e571. 31393290 10.1097/BPO.0000000000001075

[47] Xerogeanes JW , Takeda Y , Livesay GA , Ishibashi Y , Kim HS , Fu FH , et al. Effect of knee flexion on the in situ force distribution in the human anterior cruciate ligament. Knee Surg Sports Traumatol Arthrosc. 1995;3(1):9–13. 7773824 10.1007/BF01553518

[48] Yang S , Liu Y , Ma S , Ding C , Kong Z , Li H , et al. Stress and strain changes of the anterior cruciate ligament at different knee flexion angles: a three‐dimensional finite element study. J Orthop Sci. 2024;29(4):995–1002. 37407345 10.1016/j.jos.2023.05.015

