# Supplementary material for: Physeal‐sparing anterior cruciate ligament reconstruction provides better initial joint biomechanics than complete transphyseal reconstruction in an early adolescent porcine model
Source: J Exp Orthop. 2025 Oct 6;12(4):e70289. doi: 10.1002/jeo2.70289 (PMC12498133; doi:10.1002/jeo2.70289)
Supplement: Supplementary file 1 — Supporting information. [file JEO2-12-e70289-s001.pdf]

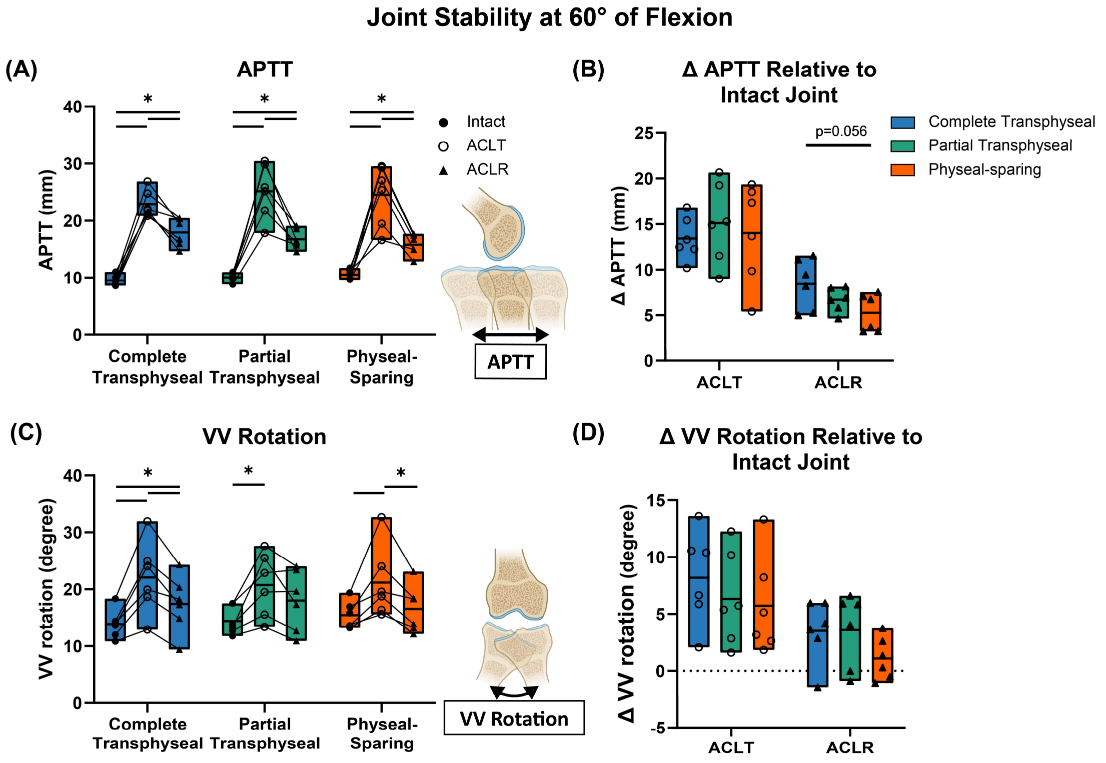

Fig. S1 Partial transphyseal and physeal-sparing technique showed similar ability to restore joint stability in early adolescent porcine joints at 60° of flexion. (A) All techniques could not restore anterior-posterior tibial translation (APTT) to intact state. (B) Increases in APTT following physeal-sparing technique were slightly smaller than those after complete transphyseal technique. (C) Varus-valgus (VV) rotation was restored to intact state when using partial transphyseal and physeal-sparing technique but not the complete transphyseal technique. (D) VV increases relative to intact state were variable across techniques. Data points presented with mean values as bars. Paired samples were connected. Statistical significance ($P < .05$) between states indicated (*).

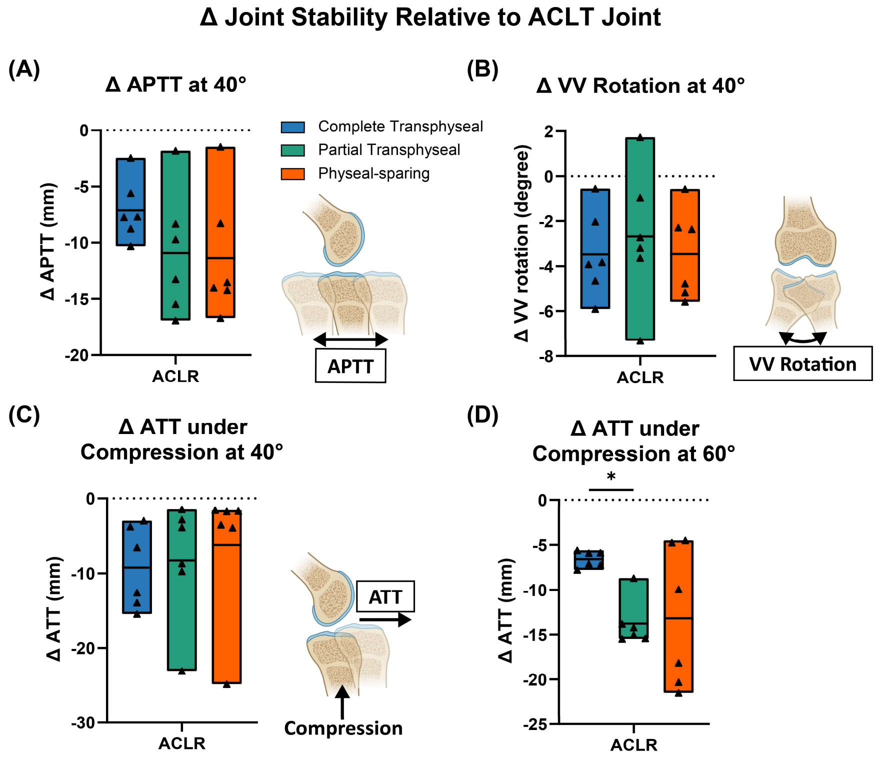

Fig. S2 Changes in joint stability following ACLR relative to ACLT joint were evaluated. At 40° of flexion, reduction of (A) APTT, (B) VV rotation, and (C) ATT under compression were similar across surgical techniques. (D) At 60° of flexion, partial transphyseal could reduce more ATT under compression compared to complete transphyseal technique. Data points presented with mean values as bars. Statistical significance ($P < .05$) between states indicated (*).

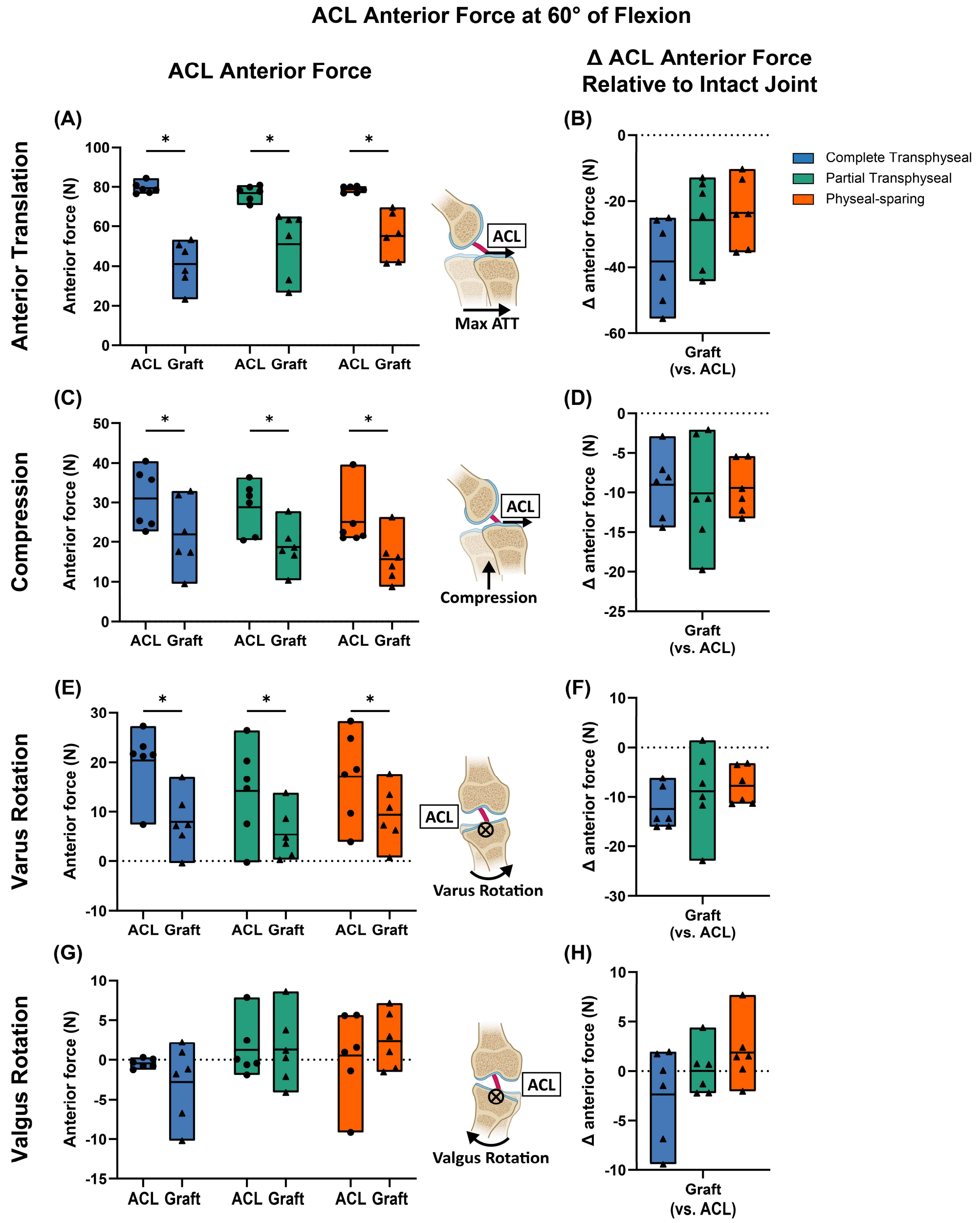

Fig. S3 Anterior forces taken by ACL decreased following ACL reconstruction at 60° of flexion. (A) Anterior forces taken by reconstructed ACL graft were not comparable to native ACL under anterior tibial translation, compression, and varus rotation, but variable under valgus rotation. (B) Decreases in anterior forces taken by ACL graft relative to native ACL were similar across techniques. ⊗ represents the ACL anterior force direction pointing into boards. Data points presented with mean values as bars. Statistical significance (P < .05) between states indicated (*).

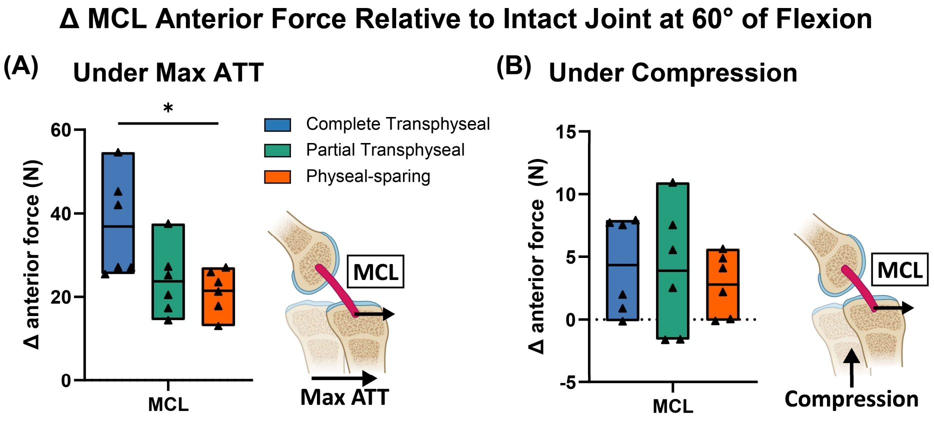

Fig. S4 Increases in MCL anterior force were higher following complete transphyseal ACLR at 60° of flexion. (A) Anterior forces under anterior translation taken by MCL increased following ACLR by different techniques. (B) More anterior forces under anterior translation shifted to MCL following complete transphyseal technique, while changes in MCL anterior forces were similar across techniques under compression. Data points presented with mean values as bars. Statistical significance ($P < .05$) between techniques indicated (*).

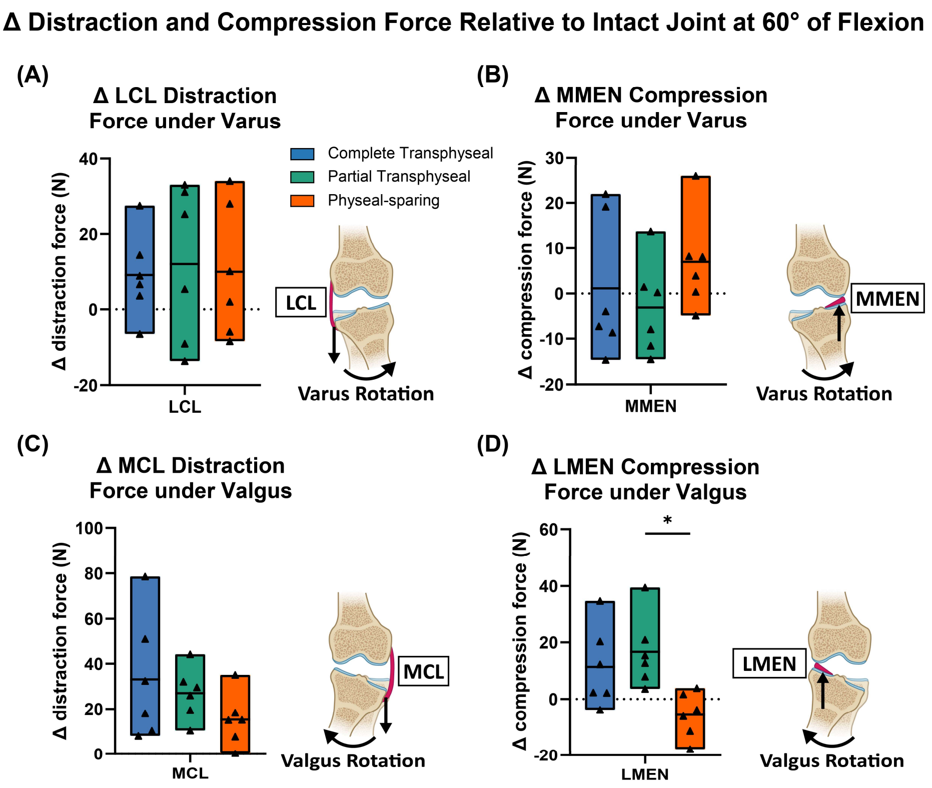

Fig. S5 Distraction forces taken by collateral ligaments as well as compression forces taken by menisci were assessed under maximum varus-valgus rotation at 60° of flexion. (A) Increases in LCL distraction forces under varus rotation were similar across techniques. (B) Compression forces on the medial meniscus (MMEN) under varus rotation showed variability, with no differences in changes following ACL reconstruction by different techniques. (C) No differences were observed in increases in MCL distraction forces under valgus rotation across techniques. (D) Compression forces on the lateral meniscus (LMEN) under valgus rotation increased following partial transphyseal ACLR but decreased following physeal-sparing ACLR. Data points presented with mean values as bars. Statistical significance ($P < .05$) between techniques indicated (*).

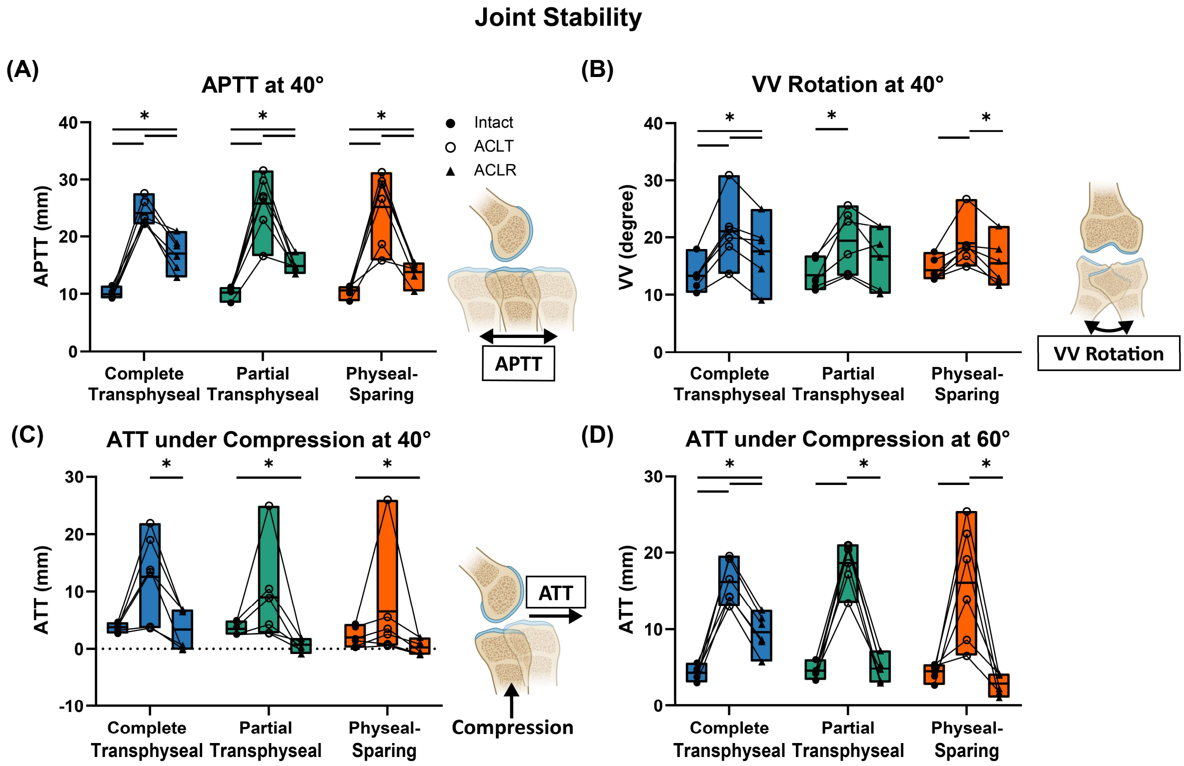

Fig. S6 Joint stability of (A) anterior-posterior tibial translation at 40°, (B) varus-valgus rotation at varus-valgus rotation at 40° of flexion, (C) anterior tibial translation under compression at 40° and (D) 60° of flexion. Paired samples were connected. Data points presented with mean values as bars. Statistical significance ($P < .05$) between techniques indicated (*).

Table S1. Detailed Robotic Testing Protocol.

Joint State	Prescribed Trajectory	Data Acquired
Intact	Passive flexion-extension	Passive path positions
	Anterior-posterior, compression, and varus-valgus loads	Intact joint kinematics (K_{int})
	Repeat K_{int}	*In situ* force of intact joint ($Joint_{int}$)
ACL Transection (ACLT)	Repeat K_{int}	*In situ* force of ACL (ACL_{int})
	Anterior-posterior, compression, and varus-valgus loads	ACLT joint kinematics (K_{ACLT})
	Repeat K_{ACLT}	*In situ* force of ACLT joint ($Joint_{ACLT}$)
ACL Reconstruction (ACLR)	Anterior-posterior, compression, and varus-valgus loads	ACLR joint kinematics (K_{ACLR})
	Repeat K_{ACLR}	*In situ* force of ACLT joint ($Joint_{ACLR}$)
Graft Transected	Repeat K_{ACLR}	*In situ* force of Graft ($Graft_{ACLR}$)
MCL Transected	Repeat K_{int}, K_{ACLT}, K_{ACLR}	*In situ* force of MCL (MCL_{int}, MCL_{ACLT}, MCL_{ACLR})
LCL Transected	Repeat K_{int}, K_{ACLT}, K_{ACLR}	*In situ* force of LCL (LCL_{int}, LCL_{ACLT}, LCL_{ACLR})
PCL Transected	Repeat K_{int}, K_{ACLT}, K_{ACLR}	*In situ* force of PCL (PCL_{int}, PCL_{ACLT}, PCL_{ACLR})
Medial Meniscus (MMEN)	Repeat K_{int}, K_{ACLT}, K_{ACLR}	*In situ* force of MMEN ($MMEN_{int}$, $MMEN_{ACLT}$, $MMEN_{ACLR}$)
Lateral Meniscus (LMEN)	Repeat K_{int}, K_{ACLT}, K_{ACLR}	*In situ* force of LMEN ($LMEN_{int}$, $LMEN_{ACLT}$, $LMEN_{ACLR}$)

Subscripts represent the kinematic state.

Table S2. Figure 2A APTT comparison in complete transphyseal group at 40° of flexion. One-way ANOVA, Tukey's Multiple Comparisons.

Tukey's multiple comparisons test	Mean Diff.	95.00% CI of diff.	Adjusted P Value
ACLT vs. Intact	-14.23	-17.32 to -11.13	<0.0001
ACLR vs. Intact	-7.132	-11.56 to -2.700	0.0078
ACLR vs. ACLT	7.093	3.447 to 10.74	0.0034

Table S3. Figure 2A APTT comparison in partial transphyseal group at 40° of flexion. One-way ANOVA, Tukey's Multiple Comparisons.

Tukey's multiple comparisons test	Mean Diff.	95.00% CI of diff.	Adjusted P Value
ACLT vs. Intact	-15.57	-21.90 to -9.244	0.0012
ACLR vs. Intact	-4.662	-6.371 to -2.953	0.0007
ACLR vs. ACLT	10.91	3.568 to 18.26	0.0109

Table S4. Figure 2A APTT comparison in physeal-sparing group at 40° of flexion. One-way ANOVA, Tukey's Multiple Comparisons.

Tukey's multiple comparisons test	Mean Diff.	95.00% CI of diff.	Adjusted P Value
ACLT vs. Intact	-14.65	-22.92 to -6.375	0.0051
ACLR vs. Intact	-3.293	-4.981 to -1.605	0.0033
ACLR vs. ACLT	11.35	3.947 to 18.76	0.0095

Table S5. Figure 2B Delta APTT comparison under ACLT state at 40° of flexion. One-way ANOVA, Tukey's Multiple Comparisons.

Tukey's multiple comparisons test	Mean Diff.	95.00% CI of diff.	Adjusted P Value
Complete transphyseal vs. Partial transphyseal	-1.350	-8.432 to 5.732	0.8747
Complete transphyseal vs. Physeal-sparing	-0.4233	-7.506 to 6.659	0.9868
Partial transphyseal vs. Physeal-sparing	0.9267	-6.156 to 8.009	0.9386

Table S6. Figure 2B Delta APTT comparison under ACLR state at 40° of flexion. One-way ANOVA, Tukey's Multiple Comparisons.

Tukey's multiple comparisons test	Mean Diff.	95.00% CI of diff.	Adjusted P Value
Complete transphyseal vs. Partial transphyseal	2.467	-0.8186 to 5.752	0.1591
Complete transphyseal vs. Physeal-sparing	3.837	0.5514 to 7.122	0.0215
Partial transphyseal vs. Physeal-sparing	1.370	-1.915 to 4.655	0.5385

Table S7. Figure 2C ATT comparison in complete transphyseal group at 40° of flexion. One-way ANOVA, Tukey's Multiple Comparisons.

Tukey's multiple comparisons test	Mean Diff.	95.00% CI of diff.	Adjusted P Value
ACLT vs. Intact	-8.817	-18.09 to 0.4604	0.0595
ACLR vs. Intact	0.3850	-3.968 to 4.738	0.9558
ACLR vs. ACLT	9.202	1.986 to 16.42	0.0202

Table S8. Figure 2C ATT comparison in partial transphyseal group at 40° of flexion. One-way ANOVA, Tukey's Multiple Comparisons.

Tukey's multiple comparisons test	Mean Diff.	95.00% CI of diff.	Adjusted P Value
ACLT vs. Intact	-5.515	-15.56 to 4.533	0.2655
ACLR vs. Intact	2.758	1.258 to 4.258	0.0043
ACLR vs. ACLT	8.273	-2.287 to 18.83	0.1095

Table S9. Figure 2C ATT comparison in physeal-sparing group at 40° of flexion. One-way ANOVA, Tukey's Multiple Comparisons.

Tukey's multiple comparisons test	Mean Diff.	95.00% CI of diff.	Adjusted P Value
ACLT vs. Intact	-4.470	-15.72 to 6.779	0.4574
ACLR vs. Intact	1.747	0.1284 to 3.365	0.0381
ACLR vs. ACLT	6.217	-5.973 to 18.41	0.3067

Table S10. Figure 2D Delta ATT comparison under ACLT state at 40° of flexion. One-way ANOVA, Tukey's Multiple Comparisons.

Tukey's multiple comparisons test	Mean Diff.	95.00% CI of diff.	Adjusted P Value
Complete transphyseal vs. Partial transphyseal	3.303	-3.792 to 10.40	0.3605
Complete transphyseal vs. Physeal-sparing	4.350	-6.436 to 15.14	0.4483
Partial transphyseal vs. Physeal-sparing	1.047	-14.30 to 16.39	0.9734

Table S11. Figure 2D Delta ATT comparison under ACLR state at 40° of flexion. One-way ANOVA, Tukey's Multiple Comparisons.

Tukey's multiple comparisons test	Mean Diff.	95.00% CI of diff.	Adjusted P Value
Complete transphyseal vs. Partial transphyseal	2.378	-0.8015 to 5.558	0.1611
Complete transphyseal vs. Physeal-sparing	1.365	-1.815 to 4.545	0.5199
Partial transphyseal vs. Physeal-sparing	-1.013	-4.193 to 2.167	0.6921

Table S12. Figure 2E VV comparison in complete transphyseal group at 40° of flexion. One-way ANOVA, Tukey's Multiple Comparisons.

Tukey's multiple comparisons test	Mean Diff.	95.00% CI of diff.	Adjusted P Value
ACLT vs. Intact	-7.705	-11.88 to -3.529	0.0043
ACLR vs. Intact	-4.233	-8.313 to -0.1539	0.0439
ACLR vs. ACLT	3.472	0.9345 to 6.009	0.0153

Table S13. Figure 2E VV comparison in partial transphyseal group at 40° of flexion. One-way ANOVA, Tukey's Multiple Comparisons.

Tukey's multiple comparisons test	Mean Diff.	95.00% CI of diff.	Adjusted P Value
ACLT vs. Intact	-5.967	-10.94 to -0.9979	0.0256
ACLR vs. Intact	-3.288	-7.394 to 0.8172	0.1026
ACLR vs. ACLT	2.678	-1.315 to 6.671	0.1675

Table S14. Figure 2E VV comparison in physeal-sparing group at 40° of flexion. One-way ANOVA, Tukey's Multiple Comparisons.

Tukey's multiple comparisons test	Mean Diff.	95.00% CI of diff.	Adjusted P Value
ACLT vs. Intact	-4.670	-8.197 to -1.143	0.0174
ACLR vs. Intact	-1.218	-3.961 to 1.524	0.3889
ACLR vs. ACLT	3.452	0.7896 to 6.114	0.0189

Table S15. Figure 2F Delta VV comparison under ACLT state at 40° of flexion. One-way ANOVA, Tukey's Multiple Comparisons.

Tukey's multiple comparisons test	Mean Diff.	95.00% CI of diff.	Adjusted P Value
Complete transphyseal vs. Partial transphyseal	1.737	-1.927 to 5.401	0.3498
Complete transphyseal vs. Physeal-sparing	3.037	-1.653 to 7.727	0.1830
Partial transphyseal vs. Physeal-sparing	1.300	-2.208 to 4.808	0.4999

Table S16. Figure 2F Delta VV comparison under ACLR state at 40° of flexion. One-way ANOVA, Tukey's Multiple Comparisons.

Tukey's multiple comparisons test	Mean Diff.	95.00% CI of diff.	Adjusted P Value
Complete transphyseal vs. Partial transphyseal	0.9433	-3.230 to 5.116	0.8290
Complete transphyseal vs. Physeal-sparing	3.015	-1.158 to 7.188	0.1796
Partial transphyseal vs. Physeal-sparing	2.072	-2.101 to 6.245	0.4223

Table S17. Figure 3A ATT comparison in complete transphyseal group at 60° of flexion. One-way ANOVA, Tukey's Multiple Comparisons.

Tukey's multiple comparisons test	Mean Diff.	95.00% CI of diff.	Adjusted P Value
ACLT vs. Intact	11.89	7.883 to 15.90	0.0005
ACLR vs. Intact	5.323	1.592 to 9.054	0.0129
ACLR vs. ACLT	-6.570	-7.754 to -5.386	<0.0001

Table S18. Figure 3A ATT comparison in partial transphyseal group at 60° of flexion. One-way ANOVA, Tukey's Multiple Comparisons.

Tukey's multiple comparisons test	Mean Diff.	95.00% CI of diff.	Adjusted P Value
ACLT vs. Intact	14.07	10.39 to17.75	0.0001
ACLR vs. Intact	0.3067	-1.057 to 1.670	0.7568
ACLR vs. ACLT	-13.77	-17.20 to -10.33	0.0001

Table S19. Figure 3A ATT comparison in physeal-sparing group at 60° of flexion. One-way ANOVA, Tukey's Multiple Comparisons.

Tukey's multiple comparisons test	Mean Diff.	95.00% CI of diff.	Adjusted P Value
ACLT vs. Intact	11.58	0.8562 to 22.30	0.0380
ACLR vs. Intact	-1.603	-3.409 to 0.2024	0.0745
ACLR vs. ACLT	-13.18	-23.50 to -2.863	0.0201

Table S20. Figure 3B Delta ATT comparison under ACLT state at 60° of flexion. One-way ANOVA, Tukey's Multiple Comparisons.

Tukey's multiple comparisons test	Mean Diff.	95.00% CI of diff.	Adjusted P Value
Complete transphyseal vs. Partial transphyseal	-2.180	-10.02 to 5.660	0.7543
Complete transphyseal vs. Physeal-sparing	0.3167	-7.523 to 8.157	0.9940
Partial transphyseal vs. Physeal-sparing	2.497	-5.343 to 10.34	0.6925

Table S21. Figure 3B Delta ATT comparison under ACLR state at 60° of flexion. One-way ANOVA, Tukey's Multiple Comparisons.

Tukey's multiple comparisons test	Mean Diff.	95.00% CI of diff.	Adjusted P Value
Complete transphyseal vs. Partial transphyseal	5.017	2.171 to 7.863	0.0010
Complete transphyseal vs. Physeal-sparing	6.930	4.084 to 9.776	<0.0001
Partial transphyseal vs. Physeal-sparing	1.913	-0.9326 to 4.759	0.2211

Table S22. Figure 4A ACL anterior force under anterior translation before and after ACLR by different surgical techniques at 40° of flexion. Paired t-test.

	Mean Diff.	95.00% CI of diff.	P Value
Complete transphyseal	-21.24	-32.99 to -9.492	0.0056
Partial transphyseal	-12.10	-21.20 to -3.006	0.0188
Physeal-sparing	-8.243	-15.27 to -1.214	0.0296

Table S23. Figure 4B Delta ACL anterior force relative to intact joint under anterior translation at 40° of flexion. One-way ANOVA, Tukey's Multiple Comparisons.

Tukey's multiple comparisons test	Mean Diff.	95.00% CI of diff.	Adjusted P Value
Complete transphyseal vs. Partial transphyseal	-9.137	-22.70 to 4.422	0.2197
Complete transphyseal vs. Physeal-sparing	-13.00	-26.56 to 0.5620	0.0612
Partial transphyseal vs. Physeal-sparing	-3.860	-17.42 to 9.699	0.7444

Table S24. Figure 4C ACL anterior force under compression before and after ACLR by different surgical techniques at 40° of flexion. Paired t-test.

	Mean Diff.	95.00% CI of diff.	P Value
Complete transphyseal	-7.437	-9.958 to -4.915	0.0006
Partial transphyseal	-3.130	-7.787 to 1.527	0.1446
Physeal-sparing	-1.925	-9.279 to 5.429	0.5308

Table S25. Figure 4D Delta ACL anterior force relative to intact joint under compression at 40° of flexion. One-way ANOVA, Tukey's Multiple Comparisons.

Tukey's multiple comparisons test	Mean Diff.	95.00% CI of diff.	Adjusted P Value
Complete transphyseal vs. Partial transphyseal	-4.307	-11.78 to 3.167	0.3202
Complete transphyseal vs. Physeal-sparing	-5.507	-12.98 to 1.967	0.1690
Partial transphyseal vs. Physeal-sparing	-1.200	-8.674 to 6.274	0.9091

Table S26. Figure 4E ACL anterior force under varus rotation before and after ACLR by different surgical techniques at 40° of flexion. Paired t-test.

	Mean Diff.	95.00% CI of diff.	P Value
Complete transphyseal	-11.14	-15.87 to -6.413	0.0018
Partial transphyseal	-7.470	-12.79 to -2.154	0.0153
Physeal-sparing	-3.775	-5.294 to -2.256	0.0014

Table S27. Figure 4F Delta ACL anterior force relative to intact joint under varus rotation at 40° of flexion. One-way ANOVA, Tukey's Multiple Comparisons.

Tukey's multiple comparisons test	Mean Diff.	95.00% CI of diff.	Adjusted P Value
Complete transphyseal vs. Partial transphyseal	-3.675	-9.677 to 2.327	0.2800
Complete transphyseal vs. Physeal-sparing	-7.368	-13.37 to -1.366	0.0158
Partial transphyseal vs. Physeal-sparing	-3.693	-9.696 to 2.309	0.2767

Table S28. Figure 4G ACL anterior force under valgus rotation before and after ACLR by different surgical techniques at 40° of flexion. Paired t-test.

	Mean Diff.	95.00% CI of diff.	P Value
Complete transphyseal	-0.006667	-3.739 to 3.725	0.9965
Partial transphyseal	-0.5033	-3.630 to 2.623	0.6961
Physeal-sparing	2.748	-1.240 to 6.736	0.1367

Table S29. Figure 4H Delta ACL anterior force relative to intact joint under valgus rotation at 40° of flexion. One-way ANOVA, Tukey's Multiple Comparisons.

Tukey's multiple comparisons test	Mean Diff.	95.00% CI of diff.	Adjusted P Value
Complete transphyseal vs. Partial transphyseal	-0.1200	-4.166 to 3.926	0.9967
Complete transphyseal vs. Physeal-sparing	-2.175	-6.221 to 1.871	0.3676
Partial transphyseal vs. Physeal-sparing	-2.055	-6.101 to 1.991	0.4066

Table S30. Figure 5A Delta MCL anterior force relative to intact joint under anterior translation at 40° of flexion. One-way ANOVA, Tukey's Multiple Comparisons.

Tukey's multiple comparisons test	Mean Diff.	95.00% CI of diff.	Adjusted P Value
Complete transphyseal vs. Partial transphyseal	10.91	-4.137 to 25.95	0.1777
Complete transphyseal vs. Physeal-sparing	15.48	0.4297 to 30.52	0.0434
Partial transphyseal vs. Physeal-sparing	4.567	-10.48 to 19.61	0.7156

Table S31. Figure 5B Delta MCL anterior force relative to intact joint under compression at 40° of flexion. One-way ANOVA, Tukey's Multiple Comparisons.

Tukey's multiple comparisons test	Mean Diff.	95.00% CI of diff.	Adjusted P Value
Complete transphyseal vs. Partial transphyseal	-3.043	-8.968 to 2.882	0.3989
Complete transphyseal vs. Physeal-sparing	-3.517	-9.442 to 2.408	0.3005
Partial transphyseal vs. Physeal-sparing	-0.4733	-6.398 to 5.452	0.9766

Table S32. Figure 6A Delta LCL distraction force relative to intact joint under varus rotation at 40° of flexion. One-way ANOVA, Tukey's Multiple Comparisons.

Tukey's multiple comparisons test	Mean Diff.	95.00% CI of diff.	Adjusted P Value
Complete transphyseal vs. Partial transphyseal	-6.528	-32.72 to 19.67	0.7967
Complete transphyseal vs. Physeal-sparing	1.782	-24.41 to 27.98	0.9830
Partial transphyseal vs. Physeal-sparing	8.310	-17.89 to 34.51	0.6944

Table S33. Figure 6B Delta medial meniscus compression force relative to intact joint under varus rotation at 40° of flexion. One-way ANOVA, Tukey's Multiple Comparisons.

Tukey's multiple comparisons test	Mean Diff.	95.00% CI of diff.	Adjusted P Value
Complete transphyseal vs. Partial transphyseal	-6.917	-26.32 to 12.49	0.6328
Complete transphyseal vs. Physeal-sparing	-18.99	-38.40 to 0.4095	0.0554
Partial transphyseal vs. Physeal-sparing	-12.08	-31.48 to 7.326	0.2693

Table S34. Figure 6C Delta MCL distraction force relative to intact joint under valgus rotation at 40° of flexion. One-way ANOVA, Tukey's Multiple Comparisons.

Tukey's multiple comparisons test	Mean Diff.	95.00% CI of diff.	Adjusted P Value
Complete transphyseal vs. Partial transphyseal	5.860	-23.37 to 35.09	0.8625
Complete transphyseal vs. Physeal-sparing	19.86	-9.372 to 49.09	0.2149
Partial transphyseal vs. Physeal-sparing	14.00	-15.23 to 43.23	0.4469

Table S35. Figure 6D Delta lateral meniscus compression force relative to intact joint under valgus rotation at 40° of flexion. One-way ANOVA, Tukey's Multiple Comparisons.

Tukey's multiple comparisons test	Mean Diff.	95.00% CI of diff.	Adjusted P Value
Complete transphyseal vs. Partial transphyseal	-10.39	-28.72 to 7.936	0.3312
Complete transphyseal vs. Physeal-sparing	12.05	-6.276 to 30.38	0.2346
Partial transphyseal vs. Physeal-sparing	22.44	4.114 to 40.77	0.0161
